# Proteasome inhibition reduces plasma cell and antibody secretion, but not angiotensin II-induced hypertension

**DOI:** 10.3389/fcvm.2023.1184982

**Published:** 2023-06-02

**Authors:** Hericka Bruna Figueiredo Galvao, Quynh Nhu Dinh, Jordyn M. Thomas, Flavia Wassef, Henry Diep, Alex Bobik, Christopher G. Sobey, Grant R. Drummond, Antony Vinh

**Affiliations:** ^1^ Department of Microbiology, Anatomy, Physiology and Pharmacology, Centre for Cardiovascular Biology and Disease Research, School of Agriculture, Biomedicine and Environment, La Trobe University, Melbourne, VIC, Australia; ^2^ Biomedicine Discovery Institute, Monash University, Melbourne, VIC, Australia; ^3^ Baker Heart and Diabetes Institute, Prahran, Australia; ^4^ Department of Immunology, Monash University, Melbourne, VIC, Australia; ^5^ Centre for Inflammatory Diseases, Monash University, Clayton, VIC, Australia

**Keywords:** hypertension, bortezomib, antibody secreting cells, immunoglobulins, angiotensin II, proteasome inhibition, plasma cells

## Abstract

**Introduction:**

Depletion of mature B cells affords protection against experimental hypertension. However, whether B cell-mediated hypertension is dependent on differentiation into antibody-secreting cells (ASCs) remains unclear. Using the proteasome inhibitor, bortezomib, the present study tested the effect of ASC reduction on angiotensin II-induced hypertension.

**Methods:**

Male C57BL6/J mice were infused with angiotensin II (0.7 mg/kg/day; s.c.) for 28 days via osmotic minipump to induce hypertension. Normotensive control mice received saline infusion. Bortezomib (750 μg/kg) or vehicle (0.1% DMSO) was administered (i.v.) 3 days prior to minipump implantation, and twice weekly thereafter. Systolic blood pressure was measured weekly using tail-cuff plethysmography. Spleen and bone marrow B1 (CD19^+^B220^−^), B2 (B220^+^CD19^+^) and ASCs (CD138^hi^Sca-1^+^Blimp-1^+^) were enumerated by flow cytometry. Serum immunoglobulins were quantified using a bead-based immunoassay.

**Results:**

Bortezomib treatment reduced splenic ASCs by ∼68% and ∼64% compared to vehicle treatment in normotensive (2.00 ± 0.30 vs. 0.64 ± 0.15 × 10^5^ cells; *n* = 10–11) and hypertensive mice (0.52 ± 0.11 vs. 0.14 ± 0.02 × 10^5^ cells; *n* = 9–11), respectively. Bone marrow ASCs were also reduced by bortezomib in both normotensive (4.75 ± 1.53 vs. 1.71 ± 0.41 × 10^3^ cells; *n* = 9–11) and hypertensive mice (4.12 ± 0.82 vs. 0.89 ± 0.18 × 10^3^ cells; *n* = 9–11). Consistent with ASC reductions, bortezomib reduced serum IgM and IgG2a in all mice. Despite these reductions in ASCs and antibody levels, bortezomib did not affect angiotensin II-induced hypertension over 28 days (vehicle: 182 ± 4 mmHg vs. bortezomib: 177 ± 7 mmHg; *n* = 9–11).

**Conclusion:**

Reductions in ASCs and circulating IgG2a and IgM did not ameliorate experimental hypertension, suggesting other immunoglobulin isotypes or B cell effector functions may promote angiotensin II-induced hypertension.

## 1. Introduction

B cells represent a major arm of the adaptive immune system. Classically, their role and that of B cell derived plasmablasts and plasma cells, is to produce antibodies/immunoglobulins that provide humoral immunity against infection. However, pathophysiological roles for immunoglobulins/antibodies have also been well documented in autoimmune and inflammatory disorders, including hypertension (1). Ebringer and Doyle demonstrated large (∼30%) increases in circulating IgG in patients with severe hypertension compared to normotensive subjects (2). Since then, increases in IgG, IgM and autoantibody titers have also been observed in patients with essential (3, 4) and malignant hypertension (5). More recently, we and others (6–9) reported that B cells are required for the development of experimental hypertension. Genetic ablation of B cells or anti-CD20-mediated neutralization of B cells blunts pressor responses in preclinical models of hypertension (6). Angiotensin II-induced experimental hypertension is associated with increases in B cell activation, plasma cell numbers and serum IgG titers (6). Although an obligatory role for B cells and an association with antibody levels has been reported in preclinical models of hypertension, whether B cell differentiation into antibody-secreting cells (ASCs) and subsequent antibody production contributes to hypertension remains unclear.

Bortezomib, a reversible, boronic acid-derived proteasome inhibitor, is a first line chemotherapeutic agent used to treat multiple myeloma, a plasma cell cancer characterized by the overproduction of antibodies (10, 11). Bortezomib targets the ubiquitin-proteasome pathway by reversibly binding to the 26S proteasome and causing apoptosis of cells with high rates of proteasome activity, which includes plasma cells (10–12). Experimentally, bortezomib treatment of mice with systemic lupus erythematosus (SLE), a chronic autoimmune disease characterized by the overproduction of autoantibodies, caused a reduction in kidney infiltrating B and T cells and glomerular immunoglobulin deposition (8). In addition, bortezomib was effective at reducing the elevated mean arterial blood pressure of SLE mice, which is a feature of the SLE model (8).

We hypothesized that depletion of ASCs and associated reductions in immunoglobulins/antibodies would reduce the severity of angiotensin II-induced hypertension. Our results show that despite a reduction in ASCs and circulating IgG2a and IgM during treatment with bortezomib, neither the angiotensin II-induced hypertension or cardiac hypertrophy were attenuated. This suggests that other immunoglobulins and/or B cell functions, such as antigen presentation and/or the secretion of pro-inflammatory cytokines, may be contributing to development of hypertension.

## 2. Materials and methods

### 2.1. Animals

The angiotensin II infusion model in male mice has been the focus of the vast majority of earlier work examining the role of the immune system in hypertension. Given our earlier work (6) showed the pharmacological and genetic depletion of B cells in male mice blunted the pressor response of angiotensin II, 8 to 14-week-old male C57BL6/J mice (*n* = 42), with initial weights of 26.9 ± 0.5 g (mean ± SEM), were obtained from the La Trobe Animal Research and Teaching Facility (Bundoora, Australia) and used in the present follow up study. Mice were housed in temperature (20°C–24°C) and humidity (40%–70%) regulated rooms with a 12-h light/dark cycle, in individually ventilated cages (IVC) with access to water and food *ad libitum*. In cases where littermates were randomly allocated to different treatment groups, they were separated into different IVC cages. The present study was conducted in accordance with the Australian Code for the Care and Use of Animals for Scientific Purposes 8th Edition 2013 (updated 2021), following ethical approval by the La Trobe University Animal Ethics Committee (AEC 16-93).

### 2.2. Randomization and blinding

Mice were randomly allocated to treatment groups using an electronic coin flip to first determine normotensive and hypertensive groups, then saline and bortezomib treatment groups. Osmotic minipumps and injection solutions were prepared by researchers who were not blind to the study. Pump implantation, i.v. administrations, data collection and statistical analyses were all performed blinded.

### 2.3. Induction of hypertension

Hypertension was induced by subcutaneous infusion of angiotensin II (0.7 mg/kg/day) for 28 days via osmotic minipumps (Alzet Model 2004, USA) as previously described (13). Briefly, each mouse was anesthetized with inhaled isoflurane (induction: 5%; maintenance: 2%–3% at 0.2–0.5 L/min,) and given an analgesic (5 mg/kg carprofen, s.c.). Minipumps were then implanted dorsally between the scapulae. Mice allocated to normotensive groups were implanted with osmotic minipumps containing 0.1% acetic acid in saline. Mice were allowed to recover and received two further doses of the analgesic 24- and 48-h post-implantation.

### 2.4. Bortezomib treatment

Bortezomib, a chemotherapeutic agent used to treat plasma cell cancers (10–12), was employed at a dose previously shown to attenuate hypertension in a murine model of systemic lupus erythematosus (8). Mice received 100 µl of either bortezomib (750 μg/kg) or its vehicle (0.1% DMSO in saline) by intravenous (i.v., tail vein) injection 3 days prior to minipump implantation and twice weekly (days 1 and 4 of each week) thereafter up to 28 days post-implantation.

### 2.5. Blood pressure measurement

Systolic blood pressure was recorded by tail cuff plethysmography using the MC4000 Multichannel system (Hatteras Instruments, USA). Mice underwent 2 training sessions 1 week prior to baseline measurements and, following minipump implantation, systolic blood pressure was recorded weekly for 28 days. Each recording session consisted of 4 independent runs with 10 cycles each, where the first run was excluded from analysis to account for acclimatization, and an average from the clear traces thereafter was obtained.

### 2.6. Post-mortem tissue analysis

Following the 28-day experimental period, mice were euthanized by CO_2_ asphyxiation followed by diaphragmatic puncture. Blood samples were collected by cardiac puncture and immediately placed on ice until further processing. Mice were then perfused with phosphate-buffered saline (PBS) and the spleen and bone marrow were harvested and prepared for flow cytometry.

### 2.7. Flow cytometry

Splenic samples were minced with scissors in 1.5 ml tubes containing PBS, then passed through 70 μm sterile cell strainers into 50 ml Falcon tubes and incubated with Red Blood Cell (RBC; 0.15 M NH_4_Cl, 0.01 M KHCO_3_, 6.0 mM EDTA, dH_2_O) lysis buffer for 5 min. Bone marrow samples were acquired by flushing the bone marrow from femoral and tibial bones with PBS over cell strainers into 50 ml Falcon tubes. Samples were then pelleted by centrifugation and resuspended in RBC lysis buffer for 5 min. Samples were then processed as in (13, 14).

Following osmotic lysis of RBCs, samples were centrifuged and resuspended in FACS buffer (0.5% bovine serum albumin (BSA; Sigma-Aldrich, USA) in PBS). Live cells were counted using trypan blue (1:1 ratio) and ∼2 million live cells in FACS buffer were loaded onto 96-well microplates. Live cells were then stained with LIVE/DEAD™ Fixable Aqua Dead Cell Stain (1:1,000 dilution; Invitrogen) for 15 min at 4°C, washed with FACS buffer, spun and resuspended in a cocktail of fluorescently labelled antibodies against cell surface markers for 25 min at 4°C, as shown in Table 1. Cell suspensions were washed, spun and permeabilized with fixation-permeabilization buffer (eBioscience™ Fixation/Permeabilization Diluent; Invitrogen) for 30 min at 4°C. The cells were then washed with permeabilization wash buffer [eBioscience™ Permeabilization Buffer (10x); Invitrogen], spun and resuspended with an intracellular fluorescent antibody mixture (Table 1) for 15 min at room temperature. Samples were then washed with permeabilization wash buffer [eBioscience™ Permeabilization Buffer Invitrogen] and resuspended in 1% formalin in FACS buffer and stored at 4°C until analysis. Flow cytometry was performed the following day using a BC CytoFLEX S flow cytometer (Beckman Coulter, USA) where ∼1 million live leukocytes were analysed.

**Table 1:** Surface and intracellular target markers used for flow cytometry.

Target	Fluorophore	Dilution
anti-CD38	PE-Cy7	1:500
anti-CD23	FITC	1:500
anti-CXCR4	PE-610	1:500
anti-Sca-1	BV605	1:500
anti-CD138	BV650	1:500
anti-CD98	A647	1:500
anti-CD19	A700	1:500
anti-B220	BV421	1:500
anti-Blimp-1 (intracellular)	PE	1:1,000

The CD19 and B220 surface markers were used for the identification of B1 and B2 B cells since B cells express CD19 from as early as the pre-B cell stage (15), however, unlike B2 B cells, B1 B cells have little to no B220 expression (16). A modified gating strategy adopted from Wilmore et al. (17) was used to determine ASC numbers as shown in Supplementary Figure S1. Briefly, ASCs were separated based on the expression of two surface proteins, Sca-1 and CD138, where the CD138^hi^Sca-1^+^ gate identifies this cell population, followed by confirmation of their phenotype by intracellular Blimp-1 expression. ASCs were further subdivided into immature plasmablasts and terminally differentiated plasma cells, where plasmablasts were CD138^hi^Sca-1^+^Blimp-1^+^B220^+^ cells and plasma cells were CD138^hi^Sca-1^+^Blimp-1^+^B220^−^ cells. Data were analysed with the FlowJo software v10.6.2 (Tree Star Inc., USA) and cell counts per spleen or bone marrow for the cell populations were exported for statistical analyses.

### 2.8. Immunoglobulin quantification and isotyping

Blood samples were allowed to rest at room temperature for 15 min prior to spinning at 2,000 × *g* for 10 min at 4°C with. The supernatant (serum) was transferred to a fresh 1.5 ml tube and immediately stored at −80°C until further analysis. Serum IgG1, IgG2a, IgG2b, IgG3, IgA and IgM were quantified using the LEGENDplex™ mouse immunoglobulin isotyping panel (BioLegend, USA), according to the manufacturer's instructions. The immunoglobulin isotypes were quantified using a BC CytoFLEX S flow cytometer (Beckman Coulter, USA) and the data were analysed using the LEGENDplex™ Data Analysis Software Suite.

### 2.9. Power calculation and sample sizes

Power calculations indicated a minimum sample size of *n *= 9 per group was required to identify a 25% mean effect change in systolic blood pressure with 80% power, a critical significance value of 5% and standard deviation of 10%. The entire experimental protocol was performed in 3 separate cohorts comprising all four groups in each cohort, amounting to a total of *n *= 9–11 per group. Differences in group sizes (*n*) arose from expected adverse events associated with angiotensin II infusion or bortezomib treatment, including aortic aneurysms (*n* = 6) and pulmonary oedema (*n* = 4), respectively. These mice were excluded from the study.

### 2.10. Data and statistical analysis

All data sets were checked for normal distribution by the D’Agostino and Pearson test and processed accordingly prior to statistical hypothesis testing. Outliers were identified with a ROUT test set to a False Discovery rate of 1%. A null hypothesis probability of less than 5% (*P* < 0.05) was considered statistically significant. All analyses were performed with GraphPad Prism v9.0.2(134) (GraphPad Software Inc., USA). Data are presented as mean ± standard error of the mean (SEM). Data sets were analysed by either a mixed effects model with Geisser-Greenhouse correction followed by Tukey's multiple comparisons test or a two-way ANOVA followed by Tukey's multiple comparisons test.

## 3. Results

### 3.1. Bortezomib reduces B cells and antibody secreting cells in the spleen and bone marrow

**Figure 1:**
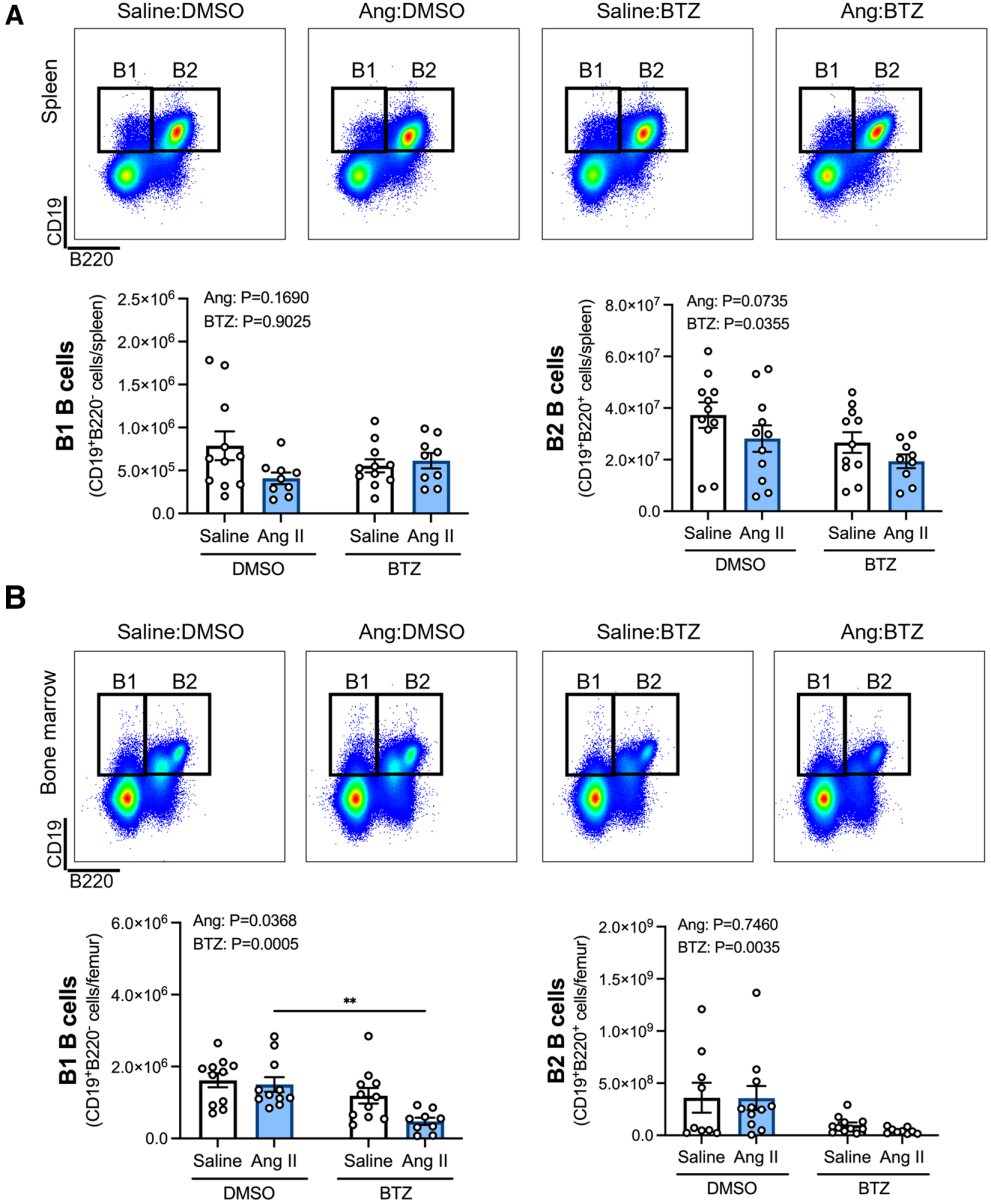
Bortezomib treatment reduces B2 B cells in the spleen and bone marrow, and B1 B cells in the bone marrow of hypertensive mice. Representative flow cytometry plots and bar graphs depicting the effect of angiotensin II and bortezomib treatment on B1 and B2 B cells in the spleen (**A**) and bone marrow (**B**) B1 (CD19^+^B220^−^) and B2 (CD19^+^B220^+^) B cells were gated from all live splenocytes and bone marrow-derived cells. Data are represented as the mean ± SEM of the total cell counts per spleen or per femur. **P value <0.01, two-way ANOVA with Tukey's multiple comparisons test. Treatment effects are indicated at the top of the bar graphs. BTZ, bortezomib.

Flow cytometry analyses of B1 (CD19^+^B220^−^) and B2 (CD19^+^B220^+^) B cells in the spleen and bone marrow revealed that whilst angiotensin II alone did not appear to affect B1 or B2 B cells in the spleen (Figure 1A), or B2 B cells in the bone marrow (Figure 1B), the combination of angiotensin II and bortezomib reduced B1 B cells in the bone marrow (∼70% reduction) (Figure 1B). Moreover, bortezomib treatment reduced B2 B cells in the spleen (∼35% reduction) (Figure 1A) and bone marrow (∼50% reduction) (Figure 1B) in both saline and angiotensin II infused mice.

**Figure 2:**
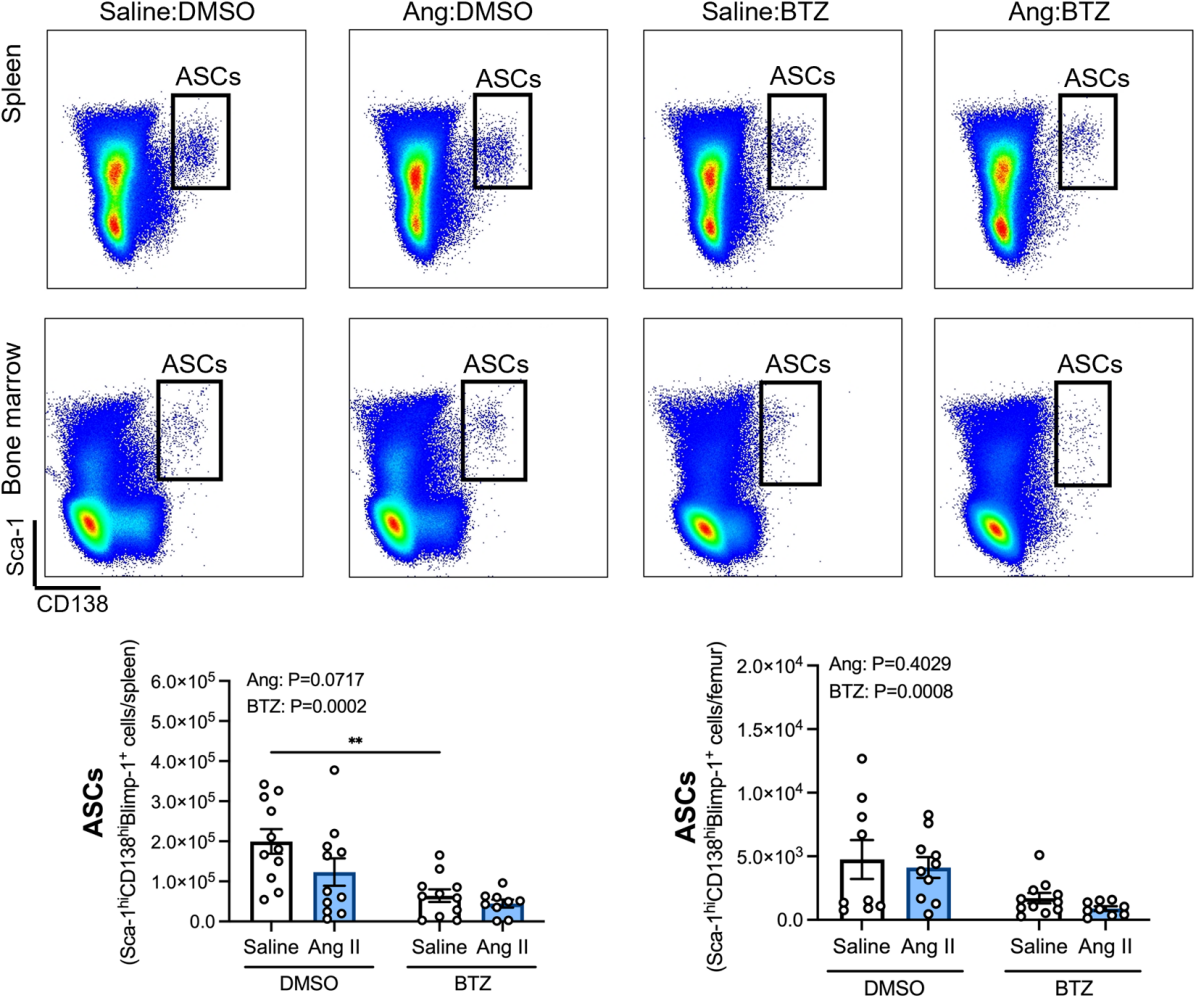
Bortezomib treatment reduces antibody secreting cells in the spleen and bone marrow. Representative flow cytometry plots and bar graphs depicting the effect of angiotensin II and bortezomib treatment on antibody secreting cells (ASCs) in the spleen and bone marrow. ASCs (CD138^hi^Sca-1^hi^Blimp-1^+^) were gated from all live splenocytes and bone marrow derived cells. Data are represented as the mean ± SEM of the total cell counts per spleen or per femur. **P value <0.01, two-way ANOVA with Tukey's multiple comparisons test. Treatment effects are indicated at the top of the bar graphs. BTZ, bortezomib.

**Figure 3:**
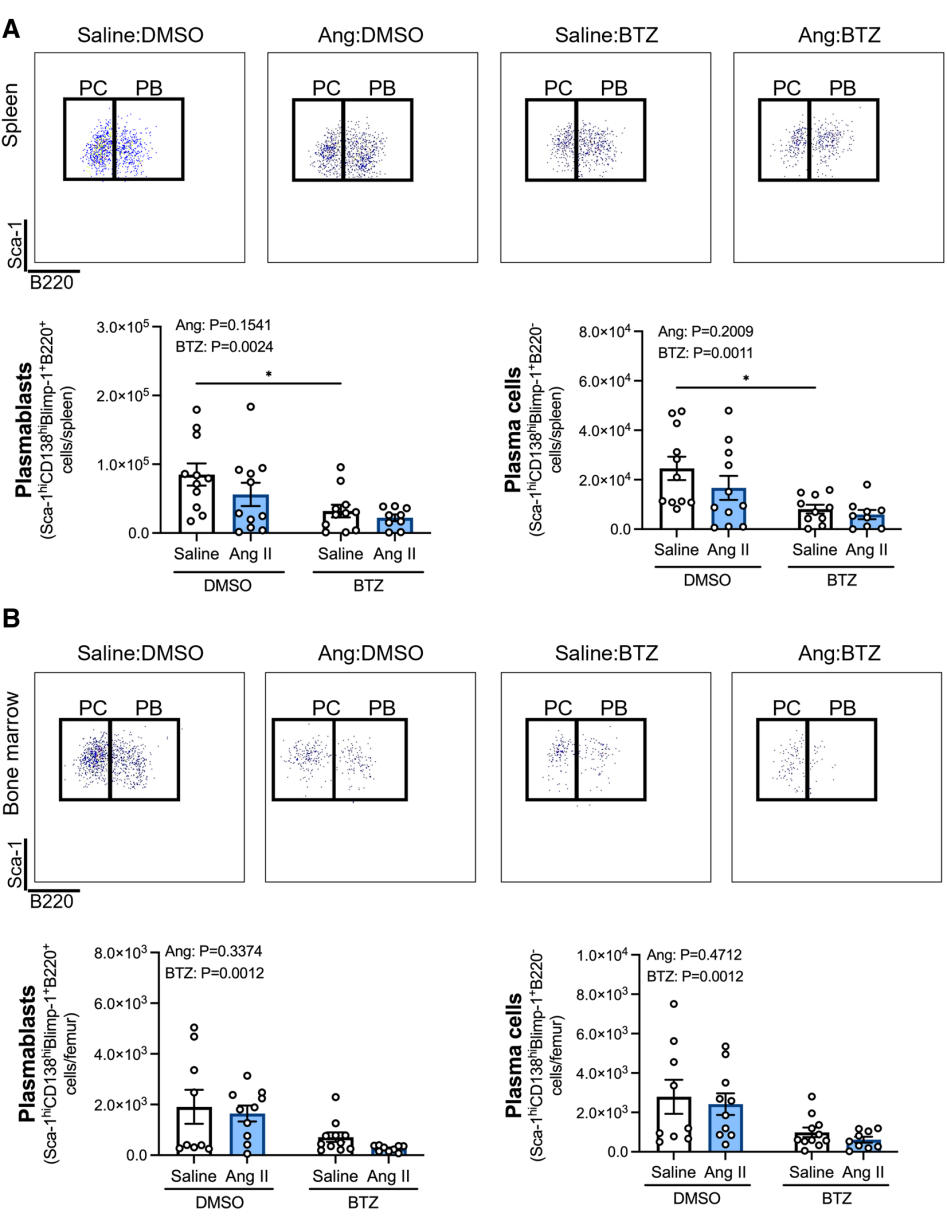
Bortezomib treatment reduces plasmablasts and plasma cells in the spleen and bone marrow. Representative flow cytometry plots and bar graphs depicting the effect of angiotensin II and bortezomib treatment on spleen (**A**) and bone marrow (**B**) plasmablasts and plasma cells. Plasmablasts (B220^+^) and Plasma cells (B220^−^) were gated from CD138^hi^Sca-1^hi^Blimp-1^+^ cells. Data are represented as the mean ± SEM of the total cell counts per spleen or per femur. *P value <0.05, two-way ANOVA with Tukey's multiple comparisons test. Treatment effects are indicated at the top of the bar graphs. BTZ, bortezomib; PC, plasma cells; PB, plasmablasts.

Angiotensin II infusion did not affect ASCs in the spleen or bone marrow (Figure 2), however, bortezomib treatment reduced ASCs by ∼66% and ∼71% compared to vehicle infused mice in the spleen and bone marrow, respectively (Figure 2). We next sought to investigate whether bortezomib preferentially affected plasmablasts (CD138^hi^Sca-1^+^Blimp-1^+^B220^+^) or plasma cells (CD138^hi^Sca-1^+^Blimp-1^+^B220^−^). As with the total number of ASCs, angiotensin II did not affect plasmablasts or plasma cells in the spleen (Figure 3A) or bone marrow (Figure 3B). However, bortezomib reduced plasmablasts by 62% and 72% in the spleen and bone marrow, respectively, compared to vehicle infused mice. Similarly, bortezomib reduced plasma cells by 66% and 69% in the spleen (Figure 3A) and bone marrow (Figure 3B), respectively. Thus, bortezomib-induced reduction of ASCs was not biased towards plasmablasts or plasma cells.

### 3.2. Bortezomib reduces circulating IgG2a and IgM

**Figure 4:**
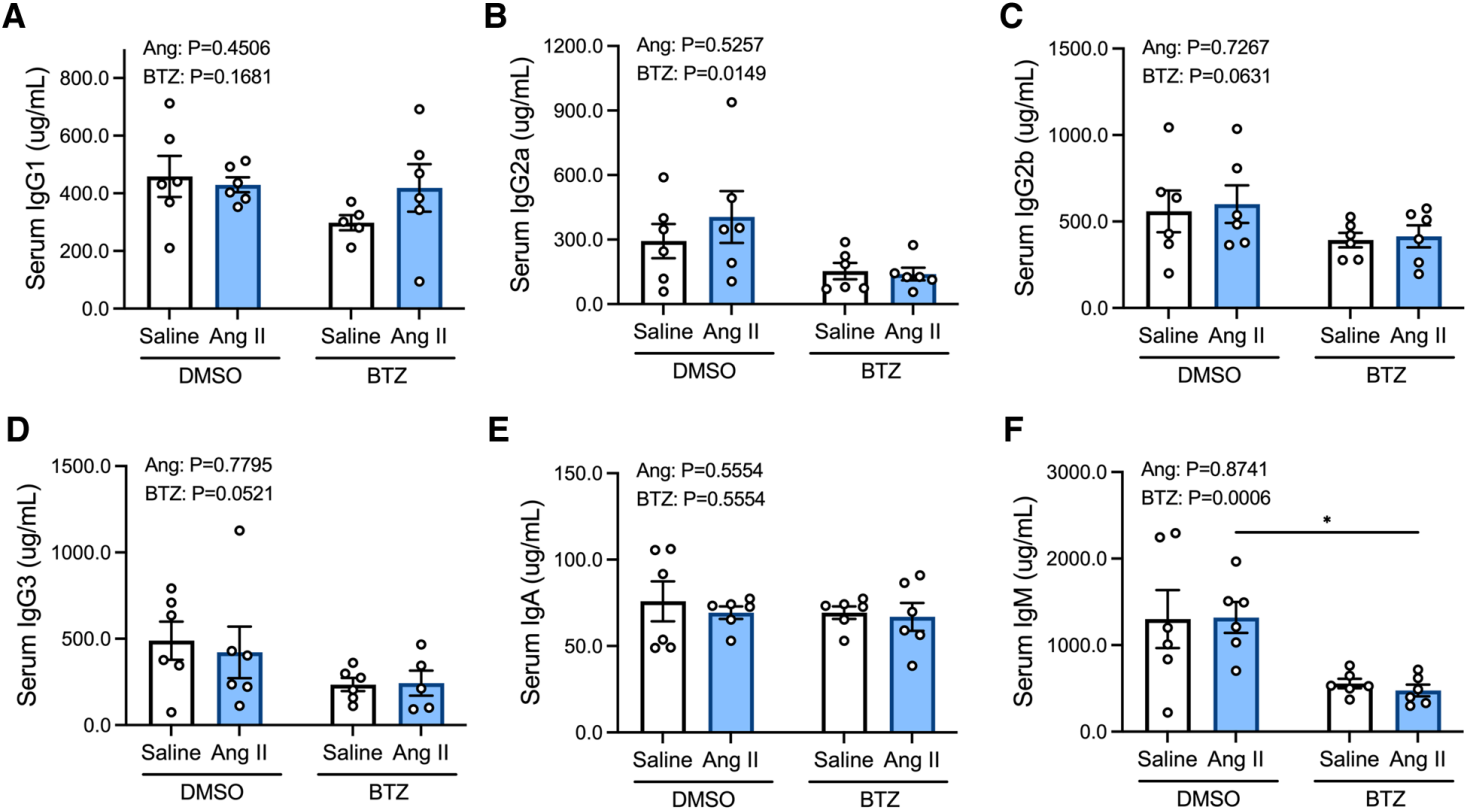
Bortezomib treatment lowers circulating IgG2a and IgM. Simultaneous quantification of mouse IgG1 (**A**), IgG2a (**B**), IgG2b (**C**), IgG3 (**D**), IgA (**E**) and IgM (**F**) immunoglobulin isotypes using a multiplex bead-based assay. Data are represented as the mean ± SEM. *P value <0.05, two-way ANOVA with Tukey's multiple comparisons test. Treatment effects are indicated at the top of the bar graphs. BTZ, bortezomib.

Angiotensin II did not affect levels of any of the immunoglobulin isotypes in the serum (Figures 4A–F); however, bortezomib reduced serum IgG2a and IgM (Figures 4B,F). There were also modest reductions in IgG2b (Figure 4C) and IgG3 (Figure 4D), which did not reach statistical significance. Serum IgG1 and IgA were unaffected.

### 3.3. Bortezomib does not attenuate increases in systolic blood pressure or cardiac hypertrophy, but increases spleen weight

**Figure 5:**
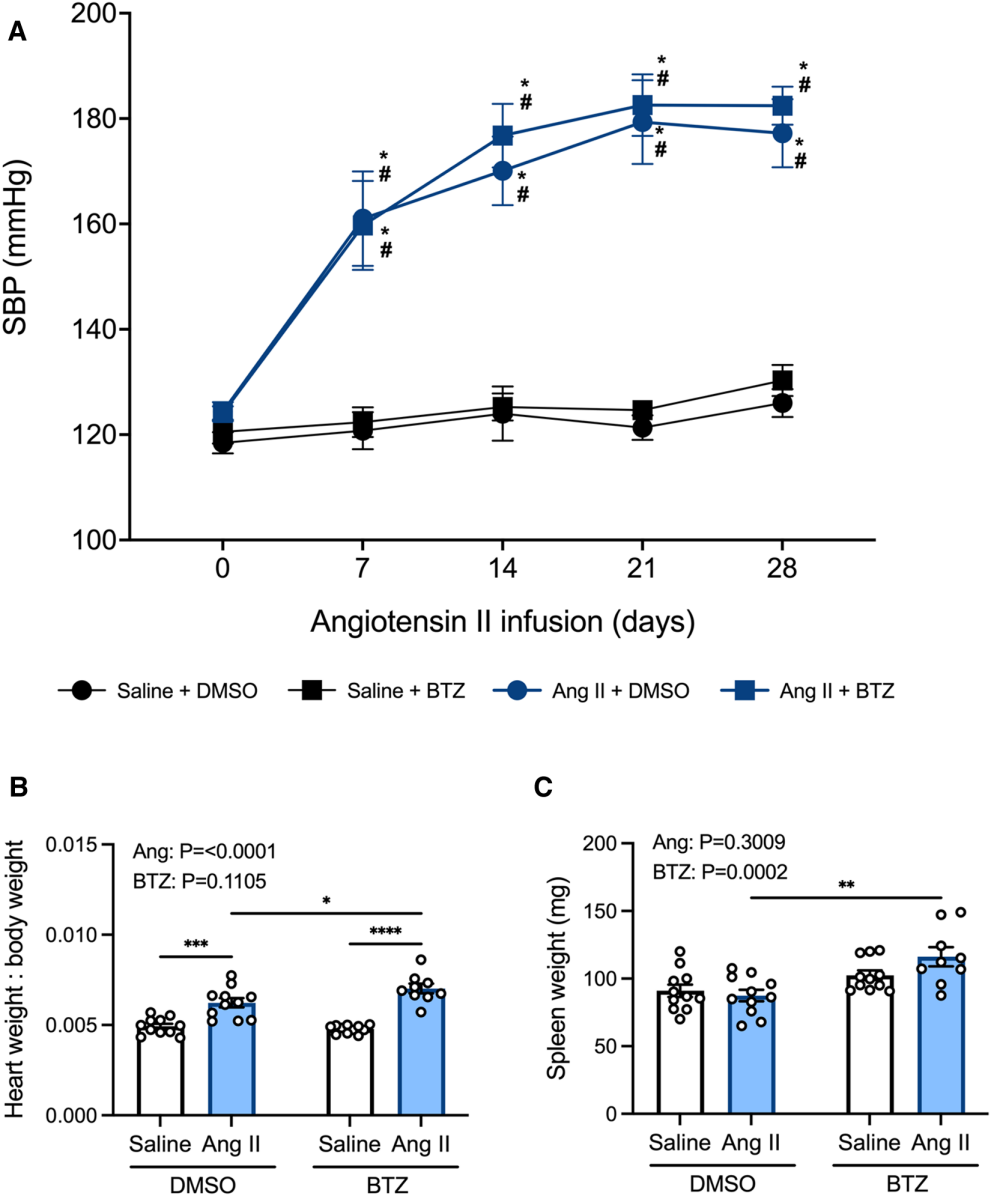
Bortezomib treatment does not prevent increases in systolic blood pressure or cardiac hypertrophy, however it increases spleen size. Systolic blood pressures (**A**), heart weight to body weight ratios (**B**), and spleen weight in mg (**C**) of saline (0.5% NaCl, 0.1% acetic acid) and angiotensin II (0.70 mg/kg/day) treated mice receiving either bortezomib (750 μg/kg; i.v.) or vehicle (0.1% DMSO). Data are represented as the mean ± SEM. For the blood pressure data * indicates a *P* value <0.05 for Ang II + vehicle vs. the saline + vehicle group; # indicates a *P* value <0.05 for Ang II + BTZ vs. the saline + BTZ group; For the heart weight to body weight and spleen weight data * indicates a *P* value <0.05, ** *P* value <0.01, ***P value <0.001 and ****P value <0.0001; Treatment effects are indicated at the top of the bar graphs. BTZ, bortezomib; SBP, systolic blood pressure.

Tail-cuff systolic blood pressure was elevated in angiotensin II infused mice from day 7 and began to plateau from day 14 (Figure 5A). Bortezomib did not prevent this increase in systolic blood pressure, including at day 28 (Ang II + vehicle: 177 ± 7 mmHg vs. Ang II + Bortezomib: 182 ± 4 mmHg; mean ± S.E.M), nor did it affect baseline systolic blood pressures of normotensive mice at day 28 (Saline + vehicle: 126 ± 3 mmHg vs. Saline + Bortezomib 130 ± 3 mmHg; mean ± S.E.M).

In normotensive mice, treatment with bortezomib had no effect on heart weight to body weight ratio (HW:BW), consistent with the lack of effect on blood pressure. Angiotensin II alone increased heart weight to body weight (HW:BW) ratio—indicative of cardiac hypertrophy (Figure 5B). Bortezomib treatment further exacerbated cardiac hypertrophy in hypertensive mice such that HW:BW was even greater than that in angiotensin II infused mice treated with vehicle (Figure 5B), however, SBPs between hypertensive mice treated with saline compared to those treated with bortezomib were not significantly different.

Prior to flow cytometry analyses, the harvested spleens were weighed to investigate potential effects of angiotensin II or bortezomib on spleen weights. Angiotensin II alone did not affect spleen weights. However, bortezomib caused an increase in spleen weights, which was more pronounced in mice treated angiotensin II (Figure 5C).

## 4. Discussion

Over the years, multiple groups have established an association between the immune system and various immune cells with the development of hypertension [reviewed in Drummond et al. (1)], with clinical evidence showing that patients with severe essential hypertension (2–4) or malignant hypertension (5) have elevated circulating immunoglobulin titers compared to normotensive individuals. Pre-clinical evidence has also shown the pharmacological and genetic depletion of B cells attenuates angiotensin II-induced hypertension (6). However, B cells can differentiate into multiple subsets which play multifaceted roles in immune signaling and thus, it is unclear whether their pro-hypertensive actions are attributable to their differentiation into ASCs and subsequent production of immunoglobulins (18). In this study, twice weekly treatment with the proteasome inhibitor bortezomib (10–12) reduced ASCs in the spleen and bone marrow, and similarly lowered serum levels of IgG2a and IgM immunoglobulins. However, these reductions did not prevent angiotensin II-induced increases in systolic blood pressure. Thus, our findings suggest that the pro-hypertensive effects of B cells may be related to other immunoglobulin isotypes or B cell functions, which could include cytokine secretion (19–26) or antigen presentation (27, 28).

To further investigate the effects of bortezomib-induced ASC reduction, ASCs were subdivided into immature plasmablasts and terminally differentiated plasma cells. Plasmablasts and plasma cells were distinguished based on their expression of the B cell surface marker B220, since plasma cells lose virtually all B cell lineage markers once terminally differentiated (29). In the present study both spleen and bone marrow plasmablasts and plasma cells were reduced. These findings corroborate previous studies where bortezomib was used to deplete splenic and bone marrow autoreactive plasma cells in mouse models of SLE. Hypertension is a feature of the model (8, 30), however, SLE-associated hypertension is underpinned by the aberrant proliferation of autoreactive ASCs and their subsequent production of autoreactive immunoglobulins which target double-stranded DNA (dsDNA) (31). In such cases, bortezomib reduced total plasma anti-dsDNA immunoglobulins, with one study reporting reductions in IgM, IgA and all IgG subclasses, with the exception of IgG2b (8, 30). While we also observed significant reductions in ASCs and some immunoglobulins following bortezomib treatment, residual ASCs and antibodies remained. Thus, it is acknowledged that residual antibody production and/or production of pro-hypertensive immunoglobulins may not have been depleted. To further investigate whether antibodies are involved in the pro-hypertensive effects of B cells, it would be interesting in future studies to determine if adoptive transfer of immunoglobulins from hypertensive mice can induce hypertension in recipient mice, or conversely whether bacterial antibody-hydrolyzing enzymes (32) can prevent angiotensin II-induced increases in systolic blood pressure.

When considering the potential role of ASCs in experimental hypertension, we previously reported increased numbers of splenic plasma cells following angiotensin II treatment (6), a finding not observed in the current study. While the expression of CD138 in mature B cells is an early sign of B cell commitment to a plasma cell fate following activation (33), developing pre-B cells may also express CD138 (34). In 2017, a new protocol for the identification of plasma cells resolved this issue without the need for reporter mice (17). Wilmore et al. (17) demonstrated that plasma cells, identified primarily by the expression of CD138, contained a population of cells which did not express the plasma cell restricted transcription factor Blimp-1, where this accounted for ∼10% of CD138^high^ cells in control C57BL6/J mice. The inclusion of pre-B cells within the CD138^high^ gate could thus result in an overestimation of the number of plasma cells and also skew the effect of conditions/interventions as multiple cell populations are found within this gate. The authors demonstrated that plasma cells are better identified by the co-expression of CD138 and Sca-1, the combination of which yielded a near 100% expression of Blimp-1^+^ cells in this gate. Thus, the discrepancy between the present study and Chan et al. (6) is likely the result of different gating strategies where Chan et al. (6) classified plasma cells as CD45^+^CD19^−^CD138^hi^ cells while here they were classified as CD138^hi^Sca-1^+^Blimp-1^+^B220^−^ cells. Of note, Wilmore et al. (17) also reported differences in the level of Sca-1 expression amongst murine strains. As such, future studies should consider the effect of angiotensin II in both mature and developing B cells, as well as which gating strategy best identifies the cells of interest for a given species/strain.

Serum immunoglobulin titers were also measured in the present study to investigate the potential downstream effects of bortezomib-induced ASC reduction. Interestingly, while Chan et al. (6) reported an increase in serum IgG titers following angiotensin II infusion, no such effect was observed in the current study. Nevertheless, bortezomib reduced IgG2a and IgM titers by ∼50%–60%. While the major discrepancies between Chan et al. (6) and the present study bring into question the role of IgGs and plasma cells in the pathogenesis of hypertension, a recent study showed that serum IgE levels were significantly upregulated in hypertensive humans and mice (35). Furthermore, IgE blockade with an anti-IgE monoclonal antibody or mast-cell specific knockout of the high affinity IgE receptor (FCεR1) reduced SBP, aortic medial wall thickening and fibrosis in angiotensin II- and DOCA/salt-induced hypertensive mice (35). Unlike other isotypes, high-affinity IgE producing cells have been shown to be short-lived plasma cells (36), that are primarily derived from the class switching of IgG1 memory B cells in mice (37). A study by Chen et al. (38) showed that angiotensin II-infused mice exhibit a significant increase in memory B cells, which upon subsequent infusion of a sub-pressor dose of angiotensin II (0.2 mg/kg/day), produced similar increases in SBPs compared to the original higher dose of angiotensin II (0.7 mg/kg/day), thus demonstrating the induction of B cell memory. Chen et al. (38) also reported that high affinity class switched antibodies are not required for the development of hypertension, however, neither the present study nor Chen et al. (38) measured serum IgE. Interestingly, bortezomib treatment did not reduce serum IgG1 titers in the present study, which may suggest B cells that are capable of class-switching into IgE-producing cells may not have been depleted. Consequently, the failure of bortezomib to reduce SBPs in the present study may reflect the persistence of pro-hypertensive, potentially IgE-producing, ASCs during the development of angiotensin II-induced hypertension, or that alternative mechanisms drive B cell-mediated hypertensive responses.

We also performed flow cytometric analysis of B1 (CD19^+^B220^−^) and B2 (CD19^+^B220^+^) B cells in the spleen and bone marrow from normotensive and hypertensive mice and observed a ∼35% reduction in splenic B2 B cells following bortezomib treatment. These results are consistent with a previous study (30) that reported a reduction in B220^+^ B cells following 1 week of bortezomib treatment in NZB/W F1 mice. In bone marrow, only 30% of B cells remained following bortezomib treatment (39). Here, we observed a ∼50% reduction in B2 B cells in both normotensive and hypertensive mice. Given B2 B cells are predominantly associated with the production of high-affinity class-switched antibodies (40), the reduction in serum IgG2a observed in this study, and the trends for reductions in IgG2b and IgG3, are in accordance with fewer B2 B cells in the spleen and bone marrow.

Interestingly, while bortezomib reduced B1 B cells in the bone marrow by less than 30% in normotensive control mice, its effect was more than doubled in angiotensin II-infused mice in which a ∼70% reduction in bone marrow derived B1 B cells was observed. Given that bortezomib targets highly active cells, the above observations may suggest that bone marrow B1 B cells from angiotensin II-infused mice were more active than those from saline infused mice. While in the present study we did not measure B cell activation, Chan et al. (6) did report an increase in expression of the activation marker CD86 on B cells in lymphoid tissues following angiotensin II infusion, thus indicating greater B cell activity in hypertensive mice. B1 B cells are primarily found in peritoneal and pleural cavities (41), and are known to migrate into the spleen and bone marrow upon activation (42) and become the main producers of “natural” serum IgM and IgA (43–45). However, B1 B cells can also class switch and produce low affinity IgG and IgE (46). Thus, the reduction in serum IgM titers observed in the present study, particularly in hypertensive mice, may suggest increased activation of IgM-producing bone marrow B1 B cell following angiotensin II infusion. However, unlike B2 B cells, bortezomib did not reduce splenic B1 B cells or serum IgA. Collectively, these results highlight the importance of considering the different B cell lineages and subtypes when investigating the potential pathogenic roles of B cells and their antibodies in disease states.

When considering the effects of proteasome inhibition in animal models of hypertension, the model chosen appears to influence the treatment outcome. In rodent models of angiotensin II-induced or SLE-associated hypertension, treatment with bortezomib at a range of doses (50, 200, 750 μg/kg) was effective at lowering mean arterial pressure (8, 47, 48). In DOCA-salt hypertensive rats, increases in systolic blood pressures were attenuated in animals treated with the proteasome inhibitor N-benzyloxycarbonyl-Ile-Glu(O-t-Bu)-Ala-leucinal (PSI) compared to vehicle (49). However, consistent with our study, bortezomib treatment over 8 weeks reduced inflammation and oxidative stress in Dahl salt sensitive rats but failed to reduce systolic blood pressure (50). These differences may be due to the pathologies associated with each animal model of hypertension, and importantly, the dosing regimen and administration of proteasome inhibitors. Furthermore, differential effects of bortezomib on cardiac hypertrophy and remodeling have also been observed. A previous study showed that angiotensin II promotes cardiac hypertrophy by upregulating the expression and activity of proteasome subunits, resulting in the degradation of angiotensin II type 1 receptor (AT1R)-associated protein, and thus potentiating AT1R-mediated p38 MAPK and STAT3 signaling (48). The same study reported that treatment with low-dose bortezomib (50 μg/kg, i.p.) three times per week for a period of 14 days reduced angiotensin II (1.4 mg/kg/day)-mediated cardiac hypertrophy and fibrosis (48). In the present study, administration of a higher dose of bortezomib (750 μg/kg, i.v.) twice weekly for 28 days potentiated the hypertrophic effects of angiotensin II (0.7 mg/kg/day), which further highlights the influence of choice of animal model and dose of bortezomib. It is also noteworthy that potential sex-specific differences remain a recurring limitation of previous studies, including our own. As such, the use of both males and females in future studies warrant consideration as much as the chosen animal model.

Bortezomib additionally caused an increase in spleen weight, particularly in angiotensin II-infused mice. This is a curious finding as 8-week treatment of NZB/W F1 mice with bortezomib reduced the SLE-associated splenomegaly (30). However, consistent with the current study, bortezomib-induced plasma cell depletion in experimental autoimmune myasthenia gravis reported increases in spleen weight following 4 or 8 weeks of bortezomib treatment (51). While the different effects of bortezomib on spleen weight may be specific to the etiology of the these autoimmune disease models, studies investigating proteasome inhibition specifically in preclinical models of hypertension (47, 49, 50), including angiotensin II infusion (48), have not reported spleen weight. In light of the observed increase in spleen weight following bortezomib treatment, our findings suggest ASCs represent a minor population in the spleen, whose non-specific reduction does not influence hypertension compared with interventions such as B cell depletion (6), splenectomy (52) or IgE blockade (35).

In conclusion, this study indicates that high dose bortezomib treatment causes a reduction in ASC numbers, in association with significant reductions in circulating IgG2a and IgM and modest reductions in IgG2b and IgG3 titers. However, neither the elevated blood pressure nor cardiac hypertrophy were affected in a mouse model of angiotensin II-induced hypertension. The findings raise the possible involvement of other immunoglobulin isotypes or other B cell functions, such as antigen presentation and/or pro-inflammatory cytokine production, in blood pressure elevations during angiotensin II-induced hypertension.

## Data Availability

The original contributions presented in the study are included in the article/**Supplementary Material**, further inquiries can be directed to the corresponding author.
