# Supplementary material for: Proteasome inhibition reduces plasma cell and antibody secretion, but not angiotensin II-induced hypertension
Source: Front Cardiovasc Med. 2023 Jun 2;10:1184982. doi: 10.3389/fcvm.2023.1184982 (PMC10272792; doi:10.3389/fcvm.2023.1184982)
Supplement: Supplementary file 1 [file Datasheet1.docx]

Supplementary Material

Proteasome inhibition reduces plasma cell and antibody secretion, but not angiotensin II induced hypertension

Hericka Bruna Figueiredo Galvao, Quynh Nhu Dinh, Jordyn Thomas, Flavia Wassef, Henry Diep, Alexander Bobik, Christopher G Sobey, Grant R Drummond^*^, Antony Vinh

*** Correspondence:** Prof Grant Drummond: [g.drummond@latrobe.edu.au](mailto:g.drummond@latrobe.edu.au)

# Supplementary Figures

**
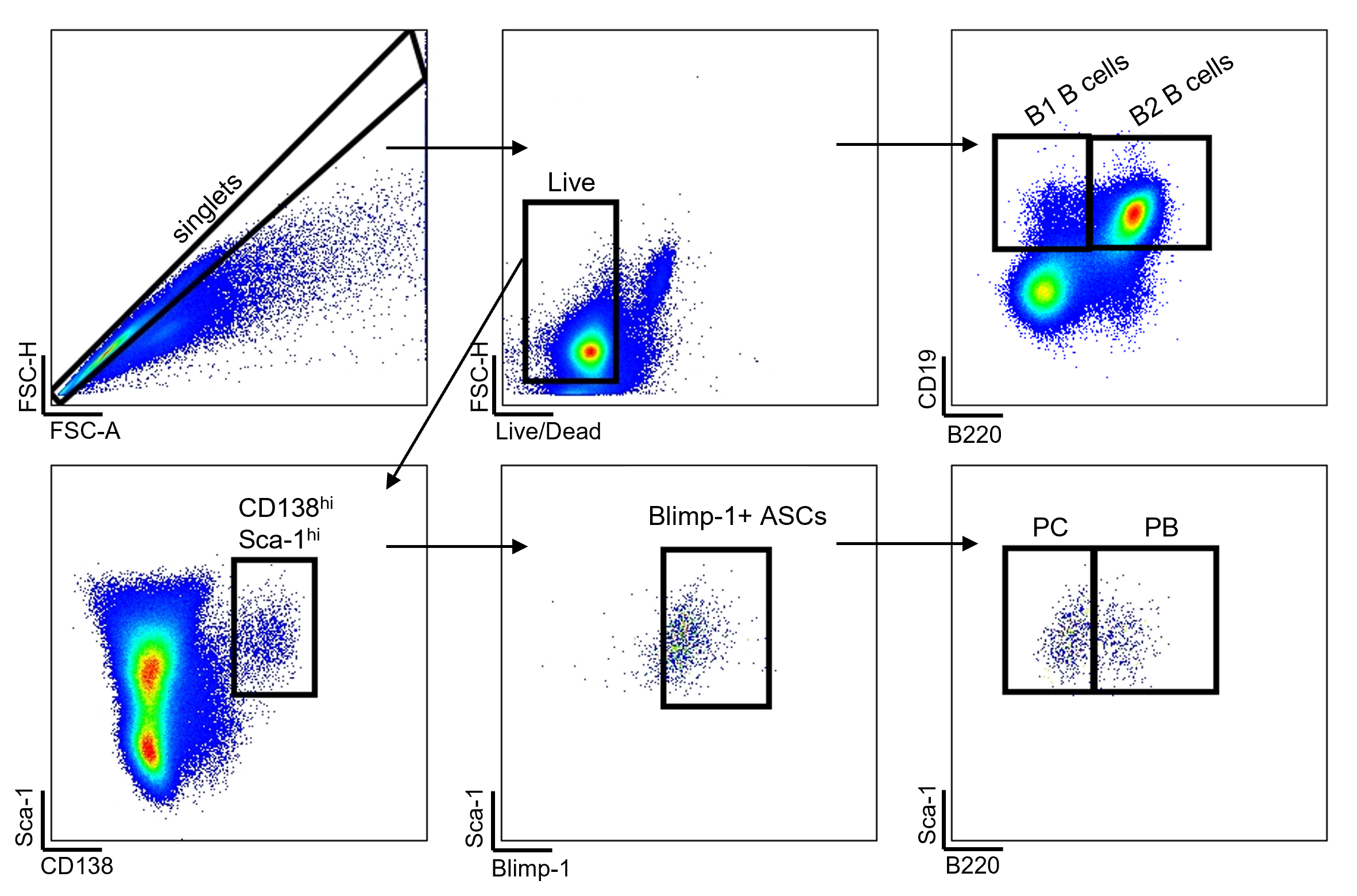
**

**Supplementary Figure 1:** **Flow cytometry gating strategy for B1 and B2 B cells, antibody secreting cells (ASCs), plasmablasts (PB) and plasma cells (PC) enumeration.**

Depiction of the gating strategy used to enumerate B1 (CD19+B220-) and B2 (CD19+B220+) B cells, ASCs (CD138hiSca-1hiBlimp-1+), PBs (CD138hiSca-1hiBlimp-1+B220+) and PCs (CD138hiSca-1hiBlimp-1+B220-) from 1% formalin fixed spleen and bone marrow samples.
